# Fn14•Trail Effectively Inhibits Hepatocellular Carcinoma Growth

**DOI:** 10.1371/journal.pone.0077050

**Published:** 2013-10-10

**Authors:** Alexandra Aronin, Shira Amsili, Tatyana B. Prigozhina, Kobi Tzdaka, Jacob Rachmilewitz, Noam Shani, Mark L. Tykocinski, Michal Dranitzki Elhalel

**Affiliations:** 1 Nephrology and Hypertension Services, Hadassah-Hebrew University Medical Center, Jerusalem, Israel; 2 Goldyne Savad Institute of Gene Therapy, Hadassah-Hebrew University Medical Center, Jerusalem, Israel; 3 KAHR Medical LTD, Jerusalem, Israel; 4 Office of the Dean, Jefferson Medical College, Philadelphia, Pennsylvania, United States of America; Osaka University Graduate School of Medicine, Japan

## Abstract

**Background:**

New strategies for the treatment of hepatocellular carcinoma (HCC) are needed, given that currently available chemotherapeutics are inefficient. Since tumor growth reflects the net balance between pro-proliferative and death signaling, agents shifting the equilibrium toward the latter are of considerable interest. The TWEAK:Fn14 signaling axis promotes tumor cell proliferation and tumor angiogenesis, while TRAIL:TRAIL-receptor (TRAIL-R) interactions selectively induce apoptosis in malignant cells. Fn14•TRAIL, a fusion protein bridging these two pathways, has the potential to inhibit tumor growth, by interfering with TWEAK:Fn14 signaling, while at the same time enforcing TRAIL:TRAIL-R-mediated apoptosis. Consequently, Fn14•TRAIL's capacity to inhibit HCC growth was tested.

**Results:**

Fn14•TRAIL induced robust apoptosis of multiple HCC cell lines, while sparing non-malignant hepatocyte cell lines. Differential susceptibility to this agent did not correlate with expression levels of TRAIL, TRAIL-R, TWEAK and Fn14 by these lines. Fn14•TRAIL was more potent than soluble TRAIL, soluble Fn14, or a combination of the two. The requirement of both of Fn14•TRAIL's molecular domains for function was established using blocking antibodies directed against each of them. Subcutaneous injection of Fn14•TRAIL abrogated HCC growth in a xenograft model, and was well tolerated by the mice.

**Conclusions:**

In this study, Fn14•TRAIL, a multifunctional fusion protein originally designed to treat autoimmunity, was shown to inhibit the growth of HCC, both *in* *vitro* and *in* *vivo*. The demonstration of this fusion protein’s potent anti-tumor activity suggests that simultaneous targeting of two signaling axes by a single fusion can serve as a basis for highly effective anti-cancer therapies.

## Introduction

Hepatocellular cancer (HCC, hepatoma) is one of the most common solid tumors, fifth in incidence worldwide [1]. Since HCC is refractory to currently available chemotherapeutics, surgical resection and liver transplantation remain the only potential curative treatment options for a limited number of suitable patients [2]. Thus, new and effective therapeutic strategies for hepatoma are needed.

The net balance of positive and negative intercellular signaling dictates tumor cell proliferation and survival, and proteins of the tumor necrosis factor (TNF) superfamily loom large in this array of regulatory signaling inputs [3]. TNF superfamily proteins contribute to tissue homeostasis via effects on cell survival, death and differentiation. In the context of HCC and various other tumor types, two members of the TNF superfamily may have special significance, namely, the cell surface ligands TWEAK (TNF-related weak inducer of apoptosis) and TRAIL (TNF-related apoptosis ligand). TWEAK is expressed either as a type 2 membrane protein (with its carboxy-terminus oriented extracellularly) or as a soluble ligand by a range of cell types, including macrophage, monocytes, NK cells, and endothelial cells [4,5]. The counter receptor for TWEAK, Fn14 (fibroblast growth –factor-inducible 14kD protein), is a membrane bound type 1 protein that is also expressed on a large variety of cells [5]. Whereas hepatocytes do not normally express Fn14 mRNA or protein, HCC cells and hepatocytes of regenerating liver express significant amounts of them [6]. 

Intriguingly, there are reports that HCC cells that have high levels of surface Fn14, also express TWEAK [7], in both membrane-anchored and soluble forms. In turn, this has led to evidence that the TWEAK:Fn14 signaling axis promotes HCC cell proliferation through autocrine and paracrine signaling [7]. Studies with glioblastoma have indicated that TWEAK induces pro-survival signaling by activating the NF-kB transcription factor and upregulating the expression of the anti-apoptotic proteins BCL-X_L_ and BCL-W. TWEAK contributes to cancer by other mechanisms as well, for example, via its pro-angiogenic activity [8,9]. 

TRAIL binds to several different cognate TNF superfamily receptors, some activating and others decoy. The activating receptors in humans are TRAIL-R1 (DR4), TRAIL-R2 (DR5), and osteoprotegrin [10,11]. There is significant cross-reactivity between the human and mice TRAIL ligands. Importantly, TRAIL selectively induces apoptosis in a range of transformed cell lines, but not in normal tissues [12,13]. In the case of HCC, there is constitutive expression of both TRAIL and its receptors [14,15]. However, the data in the literature bearing on the functionality of this TRAIL receptor are contradictory, with most data indicating that HCC cells are resistant to TRAIL-induced apoptosis. This refractoriness has suggested there may be disturbances in the apoptotic pathway and/or over-activation of pro-proliferative and survival signals in HCC cells [12,15]. 

In viewing TWEAK’s pro-proliferative and apoptosis-blockade activities in HCC, alongside the relative refractoriness of this tumor type to TRAIL-induced apoptosis, we posited that there may be functional cross-talk between these TNF pathways wherein TWEAK signaling contributes to TRAIL-resistance. In turn, this prompted the notion that blocking TWEAK:Fn14 signaling might restore death signaling through the TRAIL:TRAIL receptor axis in HCC cells. To test this hypothesis, we have here turned to Fn14•TRAIL, the recently-described fusion protein which bridges the TWEAK-Fn14 and TRAIL-TRAIL-R signaling axes [16]. In particular, the Fn14 component of Fn14•TRAIL serves to block the binding of endogenous TWEAK to Fn14 on HCC cells, while the TRAIL component, once anchored to TWEAK-bearing cells or to soluble TWEAK via the Fn14 bridge, can direct intercellular and intra-cellular inhibitory signals to its cognate receptors on TRAIL-receptor bearing cells. Thus, Fn14•TRAIL is in essence converting a TWEAK pro-proliferative signal into a death-inducing one. 

In the present study, we assess Fn14•TRAIL’s ability to induce apoptosis of HCC cell lines *in vitro* and inhibit their growth as xenograft tumors *in vivo*. Our data establish a potent anti-tumor effect of this fusion protein, and point to its considerable therapeutic potential in bridging the TWEAK-Fn14 and TRAIL-TRAIL-R signaling axes. 

## Materials and Methods

### Materials

Unless otherwise stated, all chemicals were obtained from SIGMA (Israel). DMEM medium, FBS, PBS, Trypsin-EDTA, penicillin, streptomycin and L-Glutamine were obtained from Biological Industries (Beit Haemek, Israel). 

### Cell lines

SK-HEP-1 (HTB-52; liver adenocarcinoma cell line) was purchased from ATCC (USA). HepG2, Huh7 and Hep3B HCC cell lines, originally from the ATCC, were kindly provided by the Hepatology Unit, Hadassah Hebrew University Medical Center in Jerusalem, Israel. Immortalized human hepatocyte cell lines FHB [17] and NKNT3 [18,19] were kindly provided by Prof. Eithan Galun at the Hadassah Gene Therapy Institute (Jerusalem, Israel). Cell lines were grown in 10% FBS DMEM supplemented with penicillin, streptomycin and L-Glutamine. The medium for FHB cells contained two additional components: hydrocortisone (5μM) and insulin (0.85 μM). NKNT3 cells were grown in Complete Serum-Free medium (CS-C; Cell Systems, Kyrkland, WA, USA). All cultures were tested periodically for mycoplasma contamination using EZ-PCR mycoplasma test kit (Biological Industries). 

### Protein Production

**Figure 1:**
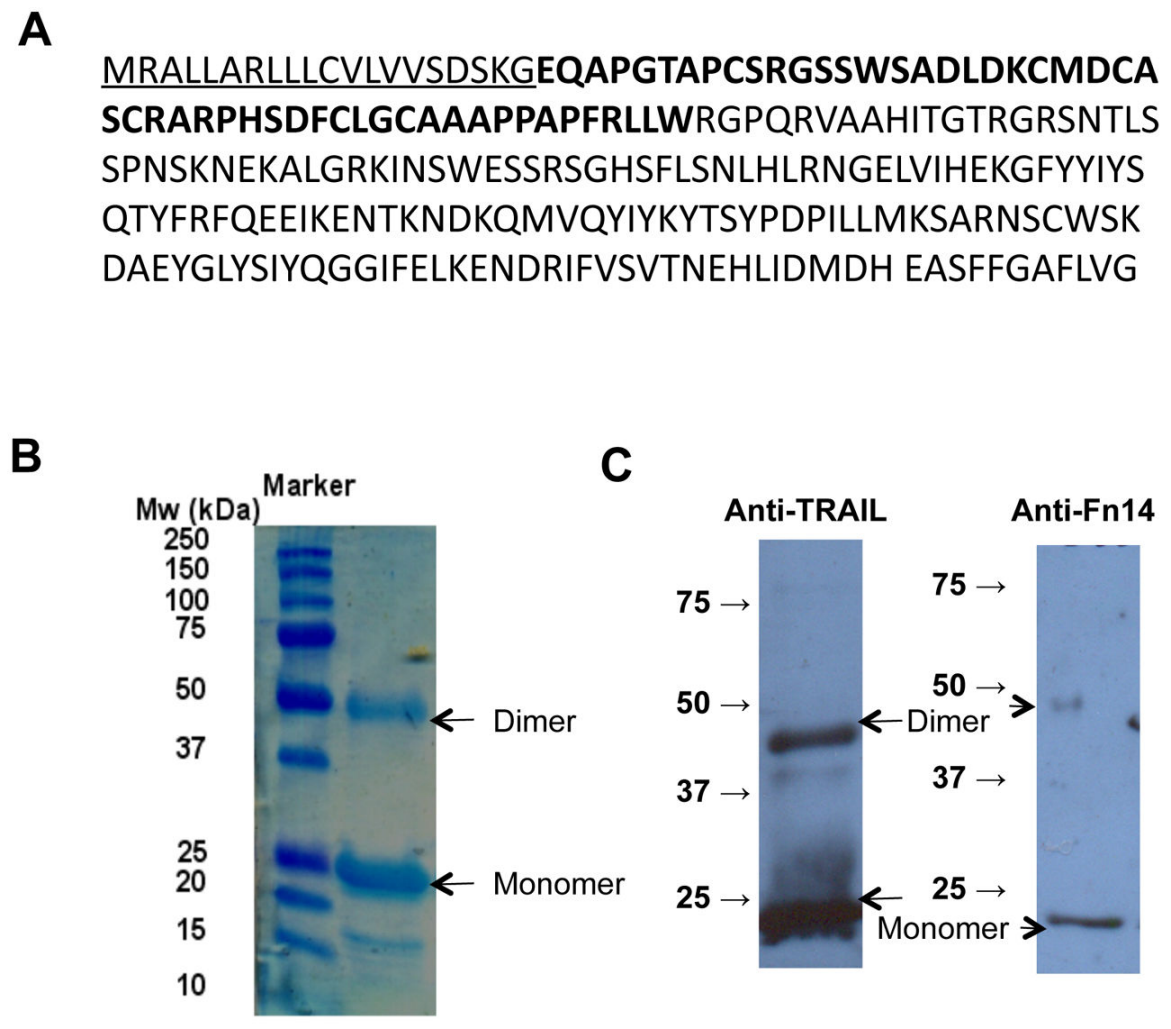
Molecular analysis of the Fn14•TRAIL. (A) The amino-acid sequence of the Fn14-TRAIL protein. The amino-acid sequence of the extra-cellular domain of human Fn14 (amino-acids 1-52 of the mature protein, marked in bold letters) are directly linked to the extra-cellular domain of human TRAIL (amino-acids 53-217 of the mature protein, non-bold letters). The underlined sequence represents the signal-peptide of the human Urokinase protein, utilized to secrete Fn14-TRAIL out of the cell and removed from the mature protein. (B) Fn14-TRAIL separated at denaturizing conditions on SDS-PAGE, Coomassie gel staining. (C) Western blot analysis with anti-TRAIL and anti-Fn14 primary antibodies.

Fn14•TRAIL was produced and purified for us by Cobra Bio-manufacturing (Keele, UK). Chinese Hamster Ovary (CHO) cells were stably transfected with a DNA construct encoding a chimeric human Fn14•TRAIL sequence downstream of the CMV promoter. A stable CHO cell transfectant clone was isolated and expanded in serum-free medium. Fn14•TRAIL was purified from the medium using chromatographic methods to over 95% purity and stored at -80°C. The amino acid sequence of the expressed Fn14•TRAIL protein is shown in Figure 1A. The underlined sequence represents the signal-peptide of the human Urokinase protein, utilized here to promote secretion of Fn14•TRAIL, it is absent from the mature protein. The amino acid sequence of the extracellular domain of human Fn14 (amino acids 1-52 of the mature protein, designated in bold letters) are directly linked to the extracellular domain of human TRAIL (amino acids 53-217 of the mature protein). 

### Western Blot and coomassie blue staining profiles of Fn14•TRAIL

Fn14•TRAIL was separated on 12% SDS–PAGE, and Coomassie blue staining was performed with GelCode blue stain reagent (PIERCE, Rockford, IL, USA), according to manufacturer instructions.

For western blot analysis, Fn14•TRAIL or whole cell lysate were separated on 10% SDS-PAGE and blotted according to standard procedures [20]. Membranes were incubated with primary antibodies: anti-TRAIL (ALEXIS biochemicals, San Diego, CA, USA), anti-Fn14 (ALEXIS biochemicals), anti Caspase 3, 8 and 9, pNFkB, Bcl-2, pJNK, cIAP1, 2(Cell Signaling), anti- IkB (R&D), and anti cFLIP (ENZO, CA, USA). Secondary immunostaining was performed with HRP-conjugated Ab (Biorad, Hercules, CA, USA). 

### Activity assay

To asses Fn14•TRAIL's activity, 0.2 x 10^6^ cells/ml (for Huh7, 0.4 x 10^6^ cells/ml) were seeded as duplicates in 24-well plates (NUNC, Roskilde, Denmark) and cultured with or without varying concentrations of Fn14•TRAIL, soluble TRAIL (hTRAIL/Apo2L; PeproTech, Rocky Hill, NJ, USA, or BioVision, Mountain View, CA USA), Fn14-Fc (recombinant mouse; ALEXIS Biochemicals, San Diego, CA, USA), or a combination of the latter two. Soluble Fn14 (recombinant human Tweak receptor, PeproTech) was used instead of Fn14-Fc in some experiments. Cells were incubated with the indicated proteins for 24h, 48h or 72h for harvested and stained with trypan-blue. Live, unstained cells were counted in a grid chamber. 

### Quantitative Real-Time PCR Analysis

Total RNA was extracted from cell lines using TRIzol reagent (Invitrogen, Carlsbad, CA, USA). For cDNA synthesis, 2 μg of total RNA were reverse-transcribed using a High Capacity cDNA Reverse Transcriptase kit (Applied Biosystems, Foster City, CA, USA), according to manufacturer's protocol. Quantitive real-time PCR was performed using TaqMan Assay-on-Demand^TM^ (TWEAK - Hs00356411_m1, Fn14 - Hs00171993_m1, TRAIL - Hs00234355_m1, DR4 - Hs00269492_m1, DR5 - Hs01043171_m1, DcR1 - Hs00182570_m1, DcR2 - Hs01043162_g1, OPG - Hs00171068_m1) and TaqMan Gene Expression Master Mix (Applied Biosystems). All reactions were performed in triplicates using the 7900HT Fast Real Time PCR system (Applied Biosystems), and normalized against two endogenous control human genes, TBP (Hs99999910_m1) and Actin-B (Hs99999903_m1). Analysis of results was based on the formula 2^-ΔΔCt^, using SDS v2.3 and data-assist v2.0 software (Applied Biosystems). 

### Flow Cytometry

To assess apoptosis, 0.2 x 10^6^ cells/ml (for Huh7, 0.4 x 10^6^ cells/ml) were seeded as duplicates in 24-well plates and incubated with or without varying concentrations of Fn14•TRAIL, soluble TRAIL, Fn14-Fc or the latter two in combination for 24 h. Apoptotic cells were detected by flow cytometric analysis, using the AnnexinV/PI MEBCYTO Apoptosis Kit (MBL, Nagoya Japan), according to the manufacturer’s protocol. 20,000 events per sample were counted using a FACSCalibur flow cytometer (Becton Dickinson, San Jose, CA, USA), and data were analyzed using CellQuest software (Becton Dickinson).

In some experiments, Fn14•TRAIL was pre-incubated with one of the following proteins, prior to addition to the cultures: anti-human/mouse Fn14 (an IgA isotype Ab); anti-hTRAIL (an IgG-1 Isotype Ab); mouse IgA and mouse IgG, with all the latter reagents obtained from eBioscience, San Diego, CA, USA. 

To examine expression of TRAIL receptors, TRAIL, Fn14 and TWEAK on cell lines, adherent cells were retrieved using Accutase (Innovative Cell Technologies, San Diego, CA, USA), washed, and stained with phycoerythrin-conjugated mAb against TWEAK (CARL-1), Fn14 (ITEM-4), TRAIL (RIK-2), the TRAIL receptors DR4 (DJR1), DR5 (DJR2-4) and DcR1 (TRAIL-R3) and DcR2 (TRAIL-R4) (R&D, Minneapolis, Minnesota, USA) or their isotype control Abs (eBioscience). Presence of intracellular TWEAK was determined using a Fixation and Permeabilization Kit (eBioscience), according to the manufacturer's protocol. Flow cytometry was performed and analyzed as above. 

### Tweak ELISA

To determine TWEAK secretion to cell culture medium, cells (10^5^) were seeded in 24 well plates for 72h and conditioned medium was collected and centrifuged. Clear medium was concentrated x3 using a speed-vac centrifuge, and analyzed by Human TWEAK ELISA development Kit (PeproTech), according to manufacturer instructions. 

### Establishment of Tumor Xenografts

All experiments were approved by the Animal Care Committee of the Hebrew University. Athymic BALB/c nu/nu nude male mice (Harlan, Israel), 4-6 weeks of age, were maintained under defined flora conditions at the Hebrew University Pathogen-Free Animal Facility. HepG2 cells were grown to 80% confluence, harvested, washed with PBS, and injected subcutaneously (4 x 10^6^/mouse) into the right flanks of mice. Once palpable, tumors were measured for their widths and lengths using a micro-caliper, and tumor volumes were calculated (w^2^ x length /2). Mice were treated daily with subcutaneous injections of Fn14•TRAIL (200 μg/mouse) for 8 days. Control groups were injected with Fn14•TRAIL dilution buffer. At the end of experiments, mice were sacrificed, and tumors were harvested, measured and weighed. 

For liver pathology assessment, Nude mice were treated for 8 days with Fn14•TRAIL and sacrificed one hour post last injection. Livers were removed, fixated in 4% formaldehyde, and embedded in paraffin. Paraffin sections (5μm) were stained with Hematoxylene and Eosin (H&E). Blood samples were taken at day 8, one hour post Fn14•TRAIL injection. Serum was kept at 4c. The next day, serum samples were diluted 1:5 or more with DDW. Aspartate aminotransferase (AST) and Alkaline phosphatase (ALK-P) serum levels were measured by the Clinical BioChemistry Analyzer Reflotron Plus Instrument and Starter Kit (Micglobal, Roche, London, UK), according to the manufacturer instructions. Serum Urea levels were determined by QuantiChromTM Urea Assay Kit (DIUR-500), (BioAssay Systems CA, USA), according to the manufacturer instructions. 

### Immunohistochemistry

To assess Fn14-TRAIL apoptosis-inducing effect *in-vivo*, HepG2 tumors bearing Athymic-Nude mice were sacrificed one hour post injection, at the 2^nd^ injection day (200ug Fn14-TRAIL per day). Subcutaneous tumors were removed, fixated in 4% formaldehyde, and embedded in paraffin. Sections (5μm) were deparaffinized in xylene (3x3') and rehydrated in graded alcohol (3x1' 100% ethanol; 3x1' 96% ethanol). Following 5' incubation in 3% H_2_O_2_ for endogenous peroxidase inactivation, slides were incubated in Citrate buffer (pH6; Invitrogen) and boiled in electric pressure cooker (BioCare Medical, CA, USA) for antigen retrieval. Samples were blocked for 30' in CAS-BLOCK (Invitrogen) prior to overnight incubation with the anti-cleaved caspase 3 primary antibody (Cell Signaling #9661; 1:100 diluted in CAS-BLOCK) at 4°c in humidified box. Following washing (3x2' in Super Sensitive wash buffer, BioGenex), samples were incubated for 30' in RT with the Simple stain MAX PO (MULTI) anti-rabbit immune-peroxidase polymer (NICHIREI BIOSCIENCES INC.). Diaminobenzidine (DAB; UltraVision Detection System, Thermo scientific, MA, USA) was used as the chromogen according to manufacturer instructions, and 20'' incubation in hematoxylene (SIGMA-Aldrich) was used as the nuclear counter-stain. Following dehydration steps (2' 80% ethanol, 2' 96% ethanol, 2' 100% ethanol, 2' xylene) and mounting (Histomount mounting solution, Invitrogen), x20 pictures were taken by the Nikon ECLIPSE Ti light microscope and captured by the DSFI-1 camera (Nikon).

### Statistical analysis

Data are presented as means ± SE. Statistical comparison of means was performed by a two-tailed unpaired Student's t test. Differences with a p<0.05 were considered statistically significant. 

## Results

### Fn14•TRAIL protein characterization

Fn14•TRAIL was produced as described in the materials and methods section. Protein molecular weight, purity and identity were verified by coommassie SDS-PAGE staining and western blot analysis with anti Fn14 and anti TRAIL antibodies (Figure 1 B and C).

### Fn14•TRAIL induces death of hepatoma cell lines

**Figure 2:**
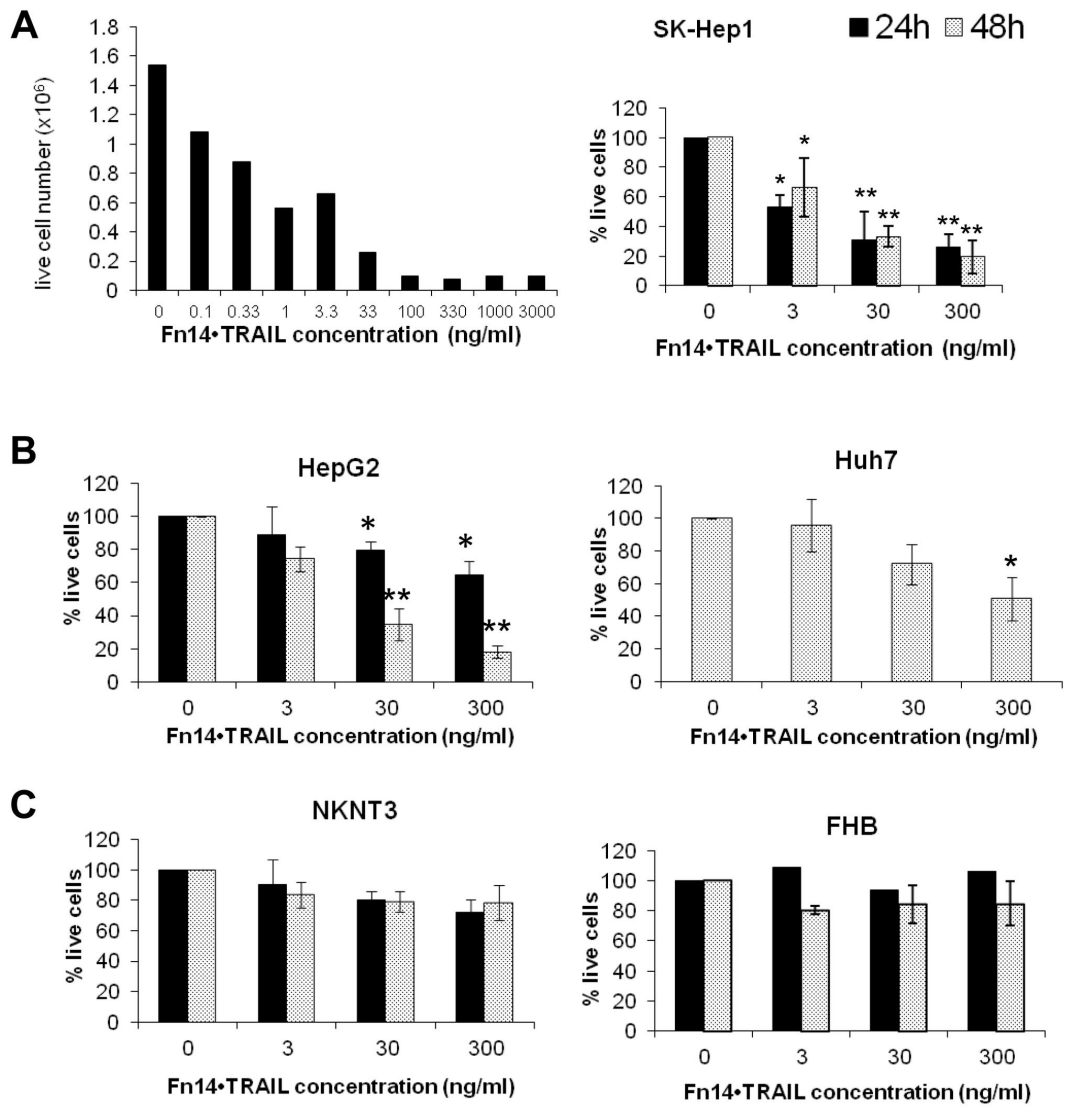
Fn14•TRAIL induces death of HCC cell lines in a dose dependent manner. SK-HEP-1 (**A**) HCC cells were incubated with indicated concentrations of Fn14•TRAIL for 72 [left panel], 48 and 24 hours [right panel]. (**B**,**C**) HepG2 [B, left panel] and Huh7 [B, right panel] HCC cell lines, as well as NKNT3 [C, left panel] and FHB [C, right panel] hepatocyte cell lines, were incubated with indicated concentrations of Fn14•TRAIL for 48 and 24 hours. Viable cells were stained with trypan blue and counted. Results represent the mean +/- SE of 3 independent experiments (* *p* < 0.05 ** *p* < 0.01 vs no Fn14•TRAIL).

To start, we evaluated Fn14•TRAIL’s cytotoxic activity against SK-HEP-1 hepatoma cells. As shown in Figure 2A, Fn14•TRAIL induces death of these cells in a dose- and time-dependent fashion. Cell death was detected at 24h and 48h, with only about 20% of live cells detectable at 30 ng/ml Fn14•TRAIL. Of note, significant cell death was detected at a concentration as low as 0.1 ng/ml, corresponding to an EC_50_ of 0.4 nmol/l. 

We extended the analysis to other hepatoma cell lines, HepG2 and Huh7. Fn14•TRAIL exhibited a cytotoxic effect against these tumor lines similar to that for SK-HEP-1 cells (Figure 2B), albeit with somewhat different kinetics. In the case of Huh7, the significant cytotoxicity was apparent only after 48 h. Importantly, non-malignant hepatocyte cell lines (NKNT3 and FHB) were resistant to death induction by Fn14•TRAIL, even at higher concentrations (Figure 2C). 

### *Fn14•*TRAIL induces apoptosis of hepatoma cell lines

**Figure 3:**
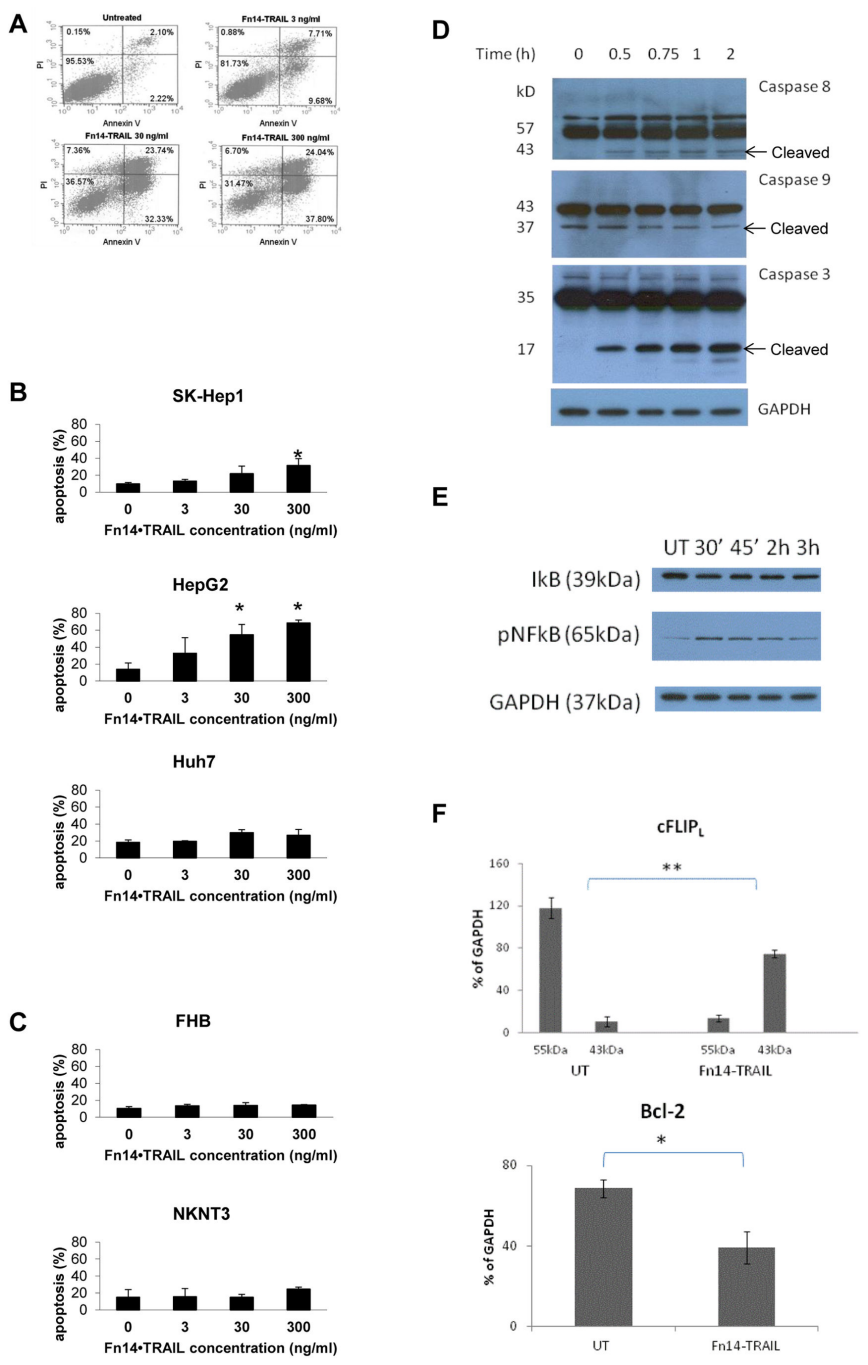
Fn14•TRAIL induces apoptosis of HCC cell lines. (**A**-**C**) SK-HEP-1 [A, and B upper panel], HepG2 [B, middle panel] and Huh7 [B, lower panel] HCC cell lines, as well as NKNT3 [C, upper panel] and FHB [C, lower panel] hepatocyte cell lines, were incubated with indicated concentrations of Fn14•TRAIL for 24 hours. Treated cells were stained by Annexin V-FITC and Propidium Iodide, and counted by flow cytometer (20,000 cells per sample). (**A**) A representative experiment, (**B**,**C**) - Results represent the mean +/- SE of 3 independent experiments (* *p* < 0.05 vs no Fn14•TRAIL). (**D**) HepG2 cells were incubated with Fn14•TRAIL for indicated time periods, whole cells lysats were immunoblotted with the indicated Abs. This is a representative out of 3 independent experiments. (**E**). HepG2 cells were incubated with Fn14•TRAIL for indicated time periods. Whole cells lysats were immunoblotted with the indicated Abs. (**F**). HepG2 cells were incubated with Fn14•TRAIL for indicated time periods. Whole cells lysats were immunoblotted with the indicated Abs. This is the summery of 3 independent experiment. The results represent the mean +/- SD of triplicates (* *p* ≤ 0.05).

Given that TRAIL is an apoptosis-inducing ligand, we next assessed Fn14•TRAIL’s capacity to induce apoptosis in malignant versus non-malignant hepatic cell lines using a standard Annexin V/PI staining assay. Early (Annexin V^+^ only) and late (Anexine V^+^ and PI^+^) apoptosis are summed together. Fn14•TRAIL effectively induced apoptosis of SK-Hep1 (Figure 3A,B), HepG2 and Huh7 cell lines in a dose-dependent manner (Figure 3B), with apoptosis apparent by the 24h time-point. Significantly, again, minimal apoptosis was detectable in the non-malignant cell lines treated with Fn14•TRAIL (Figure 3C). In order to support the notion that Fn14•TRAIL's inhibitory effect is apoptotic, caspases 3, 8, and 9 cleavage was tested using western blots. Caspases 3 and 8 were significantly cleaved already at short incubation periods (0.5-2 h) in the presence of Fn14•TRAIL (Figure 3D), and at 24 h incubation their shorter, active form appeared ( at 19 and 18 kD, respectively, not shown). Caspase 9 was spontaneously activated in this cell line, and therefore at earlier times no significant change was seen with Fn14•TRAIL treatment. (Figure 3D). Of note, experiments were repeated with all cell lines mentioned above. Caspases activation was noted only in HepG2, SK-HEP-1 and Huh7, while no such activation was present in the other cell lines that did not underwent apoptosis when incubated with Fn14•TRAIL (not shown). 

We moved to look at anti-apoptotic signaling pathways. Fn14•TRAIL was found to decrease the expression of the anti-apoptotic proteins cFLIP and Bcl-2 (Figure 3F). Similar effects of Fn14•TRAIL were observed when SK-HEP-1 cells were incubated with the protein (not shown). However, in accordance with the findings that Huh7 cells were less affected by Fn14•TRAIL, no significant changes in cFLIP or BCL-2 expression were found after the cells were cultured in the presence of Fn14•TRAIL for up to 24h. Interestingly, a decrease in IκBα levels and increase in NFkB expression was evident when cells were incubated with Fn14•TRAIL (Figure 3E). As it was previously shown that TRAIL promotes NFkB activation [21,22], this might be related to the TRAIL side of the molecule. No significant effect was found in cIAP1,2, JNK (total or phosphorilated) expression when cells were incubated with Fn14•TRAIL (not shown). 

### Variable amounts of TRAIL, TRAIL receptors, Fn14 and Tweak mRNA and protein expression levels in hepatocyte cell lines

**Figure 4:**
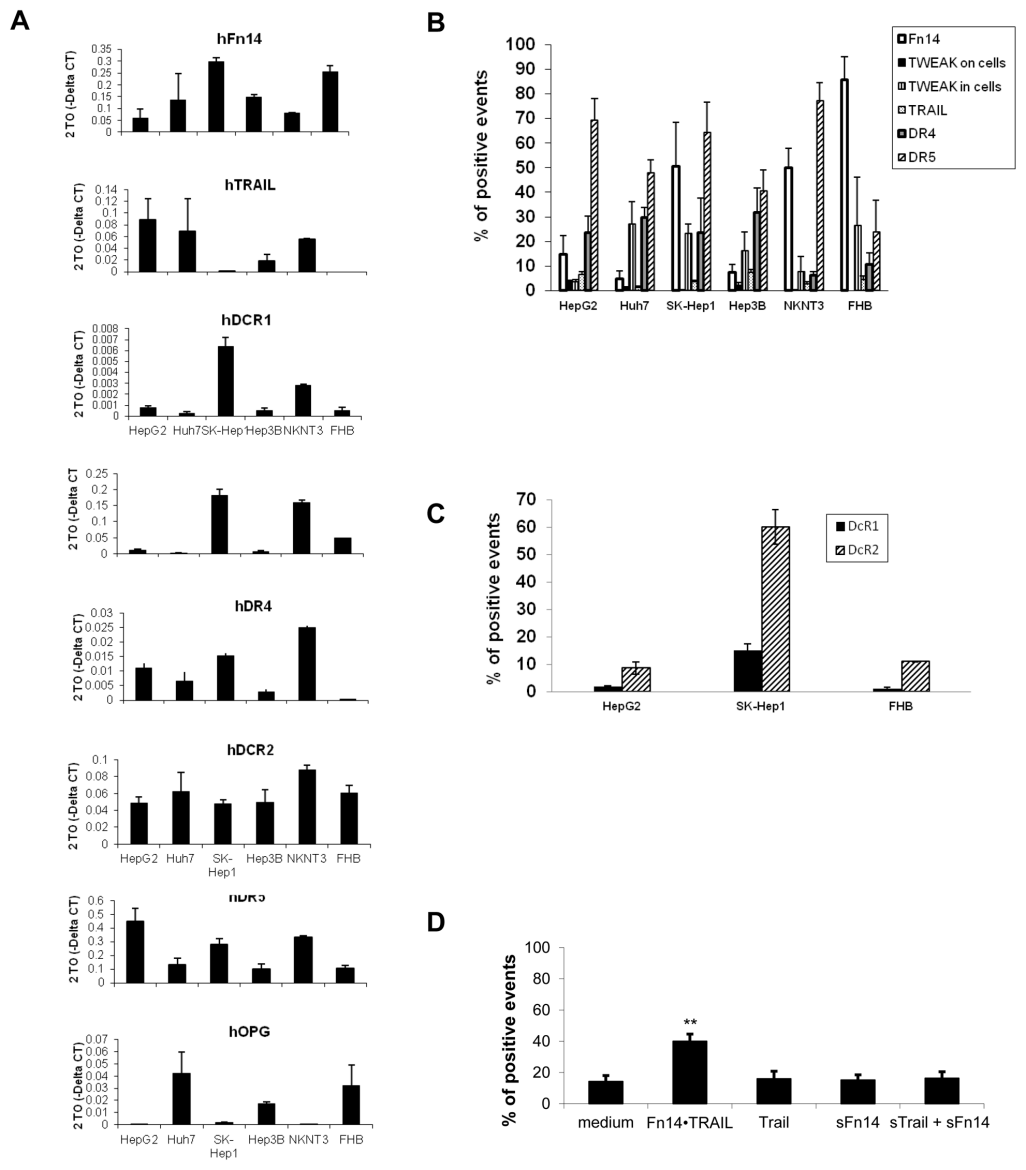
HCC and hepatocyte cell lines express TRAIL, TRAIL receptors, Fn14 and TWEAK. (**A**) The mRNA expression level of TRAIL, TRAIL receptors (DR4, DR5, DCR-1, DCR-2, OPG), Fn14 and TWEAK was determined by quantitive real-time PCR analysis. A representative experiment of three independent experiments is shown. Data are shown as average of triplicates (SD < 0.3), normalized against two endogenous control human genes, TBP and Actin-B, as calculated by Dataassist v2.0 software. (**B**,**C**) Protein expression of TRAIL, TRAIL receptors (DR4, DR5, DcR1, DcR2), Fn14 and TWEAK was determined by flow cytometeric analysis. (**D**) Fn14•TRAIL binds to HCC cells – HepG2 cells were incubated with Fn14•TRAIL, soluble TRAIL, Fn14 or the combination of the latter for 30 min at 4°c, immune-stained with PE-conjugated anti-Fn14, and analyzed by flow-cytometry. The results represent the mean +/- SD of triplicates (* *p* ≤ 0.05).

We next wanted to test whether differences in expression levels of TRAIL, Fn14, TWEAK, DR4, DR5, DcR1, DcR2 and OPG can account for the different response of the various cell lines to Fn14•TRAIL. Real time PCR analysis revealed variable mRNA expression levels of the all examined genes (Figure 4A). Interestingly, mRNA levels were not correlated with cells' sensitivity to Fn14•TRAIL, sTRAIL or Fn14, or with cell origin e.g. malignant or not. Therefore, we raised the possibility that protein expression might differ significantly between the various cell lines resulting in the observed response to Fn14•TRAIL. Cells in exponential growth were immunostained with fluorescent Abs directed against DR4, DR5, DcR1, DcR2, Fn14 and TWEAK and examined by flow cytometry. All cell lines were found to express TRAIL receptors (DR4, DR5, DcR1 and DcR2), albeit with varying surface levels (Figure 4B, C). In accordance with the real time PCR results, there was no correlation between sensitivity to Fn14•TRAIL’s apoptosis-inducing effect and the levels of surface TRAIL receptors detected by flow cytometry. While Fn14 was also expressed on all of these cell lines, its ligand, TWEAK, was present only at minimally detectable levels on the cell surfaces (Figure 4B). 

In order to examine TWEAK expression further, we proceeded to assess intracellular expression of TWEAK. Significant amounts of TWEAK were found intracellularly (Figure 4B), but TWEAK expression level did not correlate with cells' sensitivity.

Given the presence of TWEAK in the intracellular compartment, we next asked if it is secreted into the medium. To this end, cells were grown for varying time intervals up to 72 hours, and conditioned medium was analyzed by TWEAK ELISA. Secreted TWEAK was detectable in these cell cultures at low levels (not shown). Of note, again, no correlation was found between TWEAK levels and sensitivity to Fn14•TRAIL. 

### *Fn14*•TRAIL binds to hepatoma cells

We next determined Fn14•TRAIL’s ability to bind to cell surfaces. To that end, hepatoma cells were incubated in the presence or absence of Fn14•TRAIL, at 4°C prior to immunostaining with fluorescent-labeled anti-Fn14 Ab. All cell lines exhibited significant Fn14 immunostaining, consistent with Fn14•TRAIL binding to these cells (Figure 4D). This finding suggests that the differential sensitivity of non-malignant and malignant hepatic cell lines to Fn14•TRAIL is not simply explained by differences in Fn14•TRAIL binding to the respective cell types. 

### *Fn14*•TRAIL is more potent than soluble forms of TRAIL and Fn14, alone or in combination

**Figure 5:**
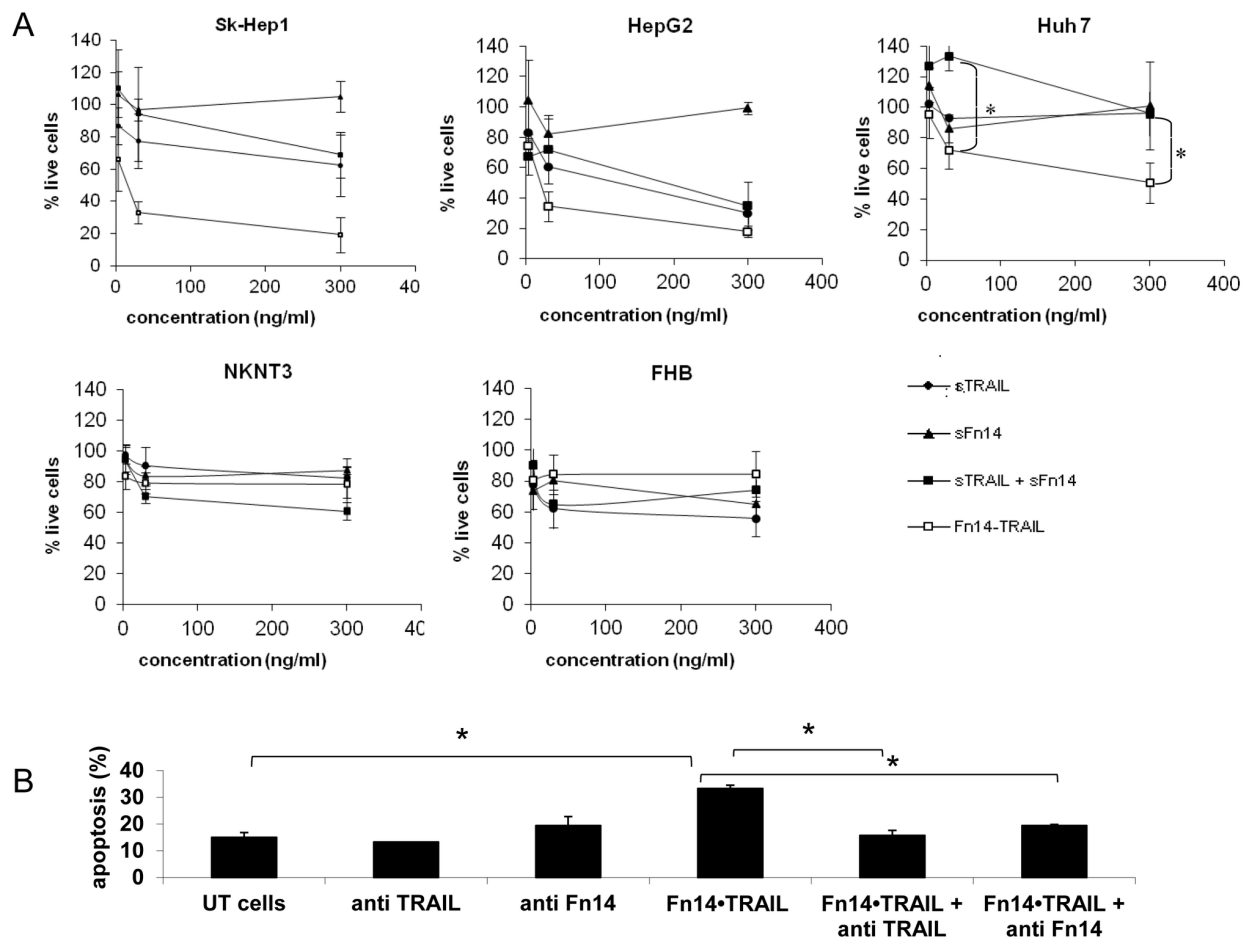
Fn14•TRAIL is more potent than soluble forms of Trail and Fn14 alone or in combination. (**A**) SK-HEP-1 [A, left panel], HepG2 [A, middle panel] and Huh7 [A, right panel] HCC cell lines, as well as NKNT3 [B, left panel] and FHB [B, right panel] hepatocyte cell lines, were incubated with 0, 3, 30 or 300 ng/ml of Fn14•TRAIL, TRAIL, Fn14-Fc or combination of the later two for 48 hours. Viable cells were stained with trypan blue and counted. The results represent the mean +/- SD of three independent experiments (* *p* ≤ 0.05). (**B**) HepG2 HCC cells were incubated with 30ng/ml Fn14•TRAIL for 24 hours, in the presence or absence of anti Fn14 or anti TRAIL blocking antibodies. Treated cells were stained by Annexin V-FITC and Propidium Iodide, and counted by flow cytometer (2x10^4^ cells per sample). The results represent the mean +/- SD of two independent experiments (* p ≤ 0.05).

We next asked whether the robust efficacy of Fn14•TRAIL as an apoptosis-inducer can be achieved by simply deploying its component parts (Fn14 and TRAIL), alone or in combination. To address this question, the various hepatic cell lines were incubated in the presence or absence of either Fn14•TRAIL, soluble TRAIL (sTRAIL), soluble Fn14 (or Fn14-Fc), or the latter two in combination. sTRAIL is composed of 168 amino-acids of the carboxy-terminal of the extracellular domain of TRAIL (19.6 kD), and differs just in the 3 first amino acids in the amino side from the TRAIL domain of Fn14•TRAIL. sFN14 (5.6 kD), is composed of the 53 amino acids of the extracellular domain of Fn14, and differs from the Fn14 part of Fn14•TRAIL by one amino acid, the first from the carboxy-side. Fn14•TRAIL molecular weight is calculated to be 24.6 kD. As shown in Figure 5, Fn14•TRAIL was substantially more efficient in inducing HCC cell death when compared not only to sTrail and Fn14-Fc, but also to a combination of the two. Of note, we repeated this set of experiments with soluble Fn14, instead of Fn14-Fc, as the later is a dimer and does not tend to form trimers as TNF-family proteins do, and the results were the same. Importantly, although comparison was made for the same dose of proteins, Fn14•TRAIL's molecular weight is heavier than that of sTRAIL or sFn14, and therefore there was actually more of the other proteins if the molar amount is considered, making the difference in efficacy more significant. 

### *Fn14*•TRAIL's activity can be blocked using anti-TRAIL and anti-Fn14 Ab

To investigate the importance of the Fn14•TRAIL fusion protein’s two ends, we turned to Ab blocking. Specifically, we incubated the HCC cell lines with Fn14•TRAIL in the presence or absence of anti-TRAIL or anti-Fn14 Ab for 24h, and assessed apoptosis by Annexine/PI staining and flow cytometry. Significantly, both anti-TRAIL and anti-Fn14 Ab completely abrogated the apoptosis induced by Fn14•TRAIL (Figure 5B), indicating that both molecular domains of the fusion protein are critical for its activity. 

### *Fn14*•TRAIL inhibits tumor growth in vivo

**Figure 6:**
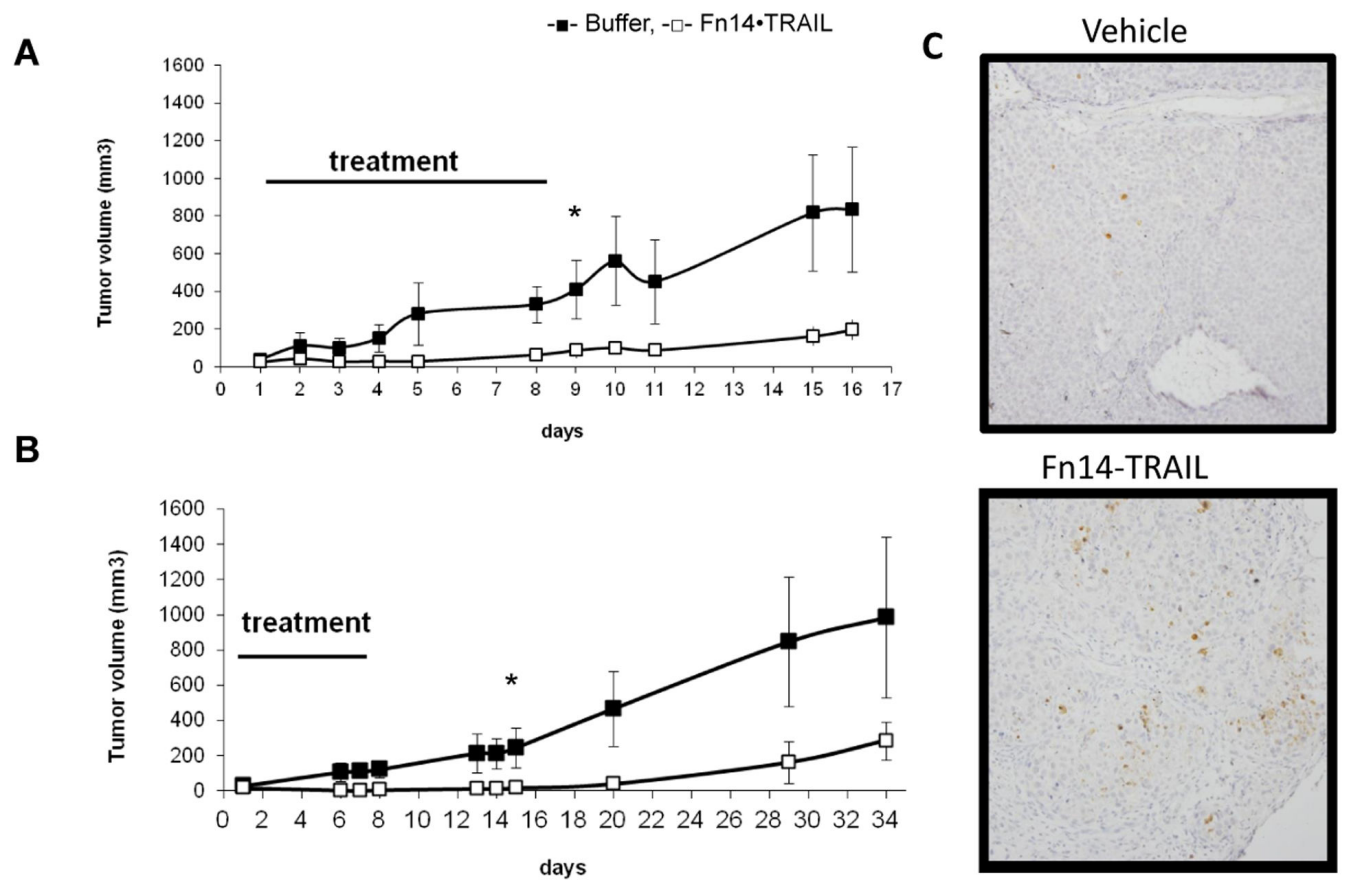
Fn14•TRAIL inhibits tumor growth in-vivo. HepG2 cells were injected subcutaneously (4-5x10^6^/mouse) to NUDE mice. Mice were treated daily with subcutaneous injections of Fn14•TRAIL (200μg) or vehicle for 8 days. Tumor volume was followed in two independent experiments, for 16 days (**A**) or 34 days (**B**). The results represent the average volume of tumors, measured for five Fn14•TRAIL treated animals and four controls (**A**), and of eight Fn14•TRAIL treated animals and seven controls (**B**), +/- SD, (* *p* ≤ 0.05) (**C**) HepG2 cells were injected subcutaneously (4-5x10^6^/mouse) to NUDE mice. Mice were treated with subcutaneous injections of Fn14•TRAIL (200μg) or vehicle for 2 days. Tumors were excised one hour after the second injection. Immunostaining was performed with anti activated caspase 3 Abs.

We next moved *in vivo*, examining Fn14•TRAIL's ability to inhibit tumor growth. NUDE mice were injected subcutaneously with HepG2 cells and followed daily for tumor growth. When tumors were palpable in individual mice, they were treated with either Fn14•TRAIL (200 µg/day) or vehicle, both administered subcutaneously once daily, for 8 consecutive days. Starting on day 2 after the initiation of treatment, a clear difference in tumor growth rate became apparent, with tumors in the Fn14•TRAIL treatment group growing slower or even decreasing in size (Figure 6A,B). Interestingly, the inhibitory effect on tumor growth persisted for at least 2-3 weeks after the 8 day treatment window. Tumors were excised and weighed at the end. The weights of excised tumors were consistent with the external measurements. In order to verify that Fn14•TRAIL induces apoptosis *in vivo*, in a different experiment, tumors from Fn14•TRAIL treated mice or vehicle treated mice were excised after two doses of treatment and were immuno-stained against cleaved caspase 3. Tumors taken from Fn14•TRAIL treated mice were stained positively, indicating that cells are undergoing apoptosis (Figure 6C). 

**Figure 7:**
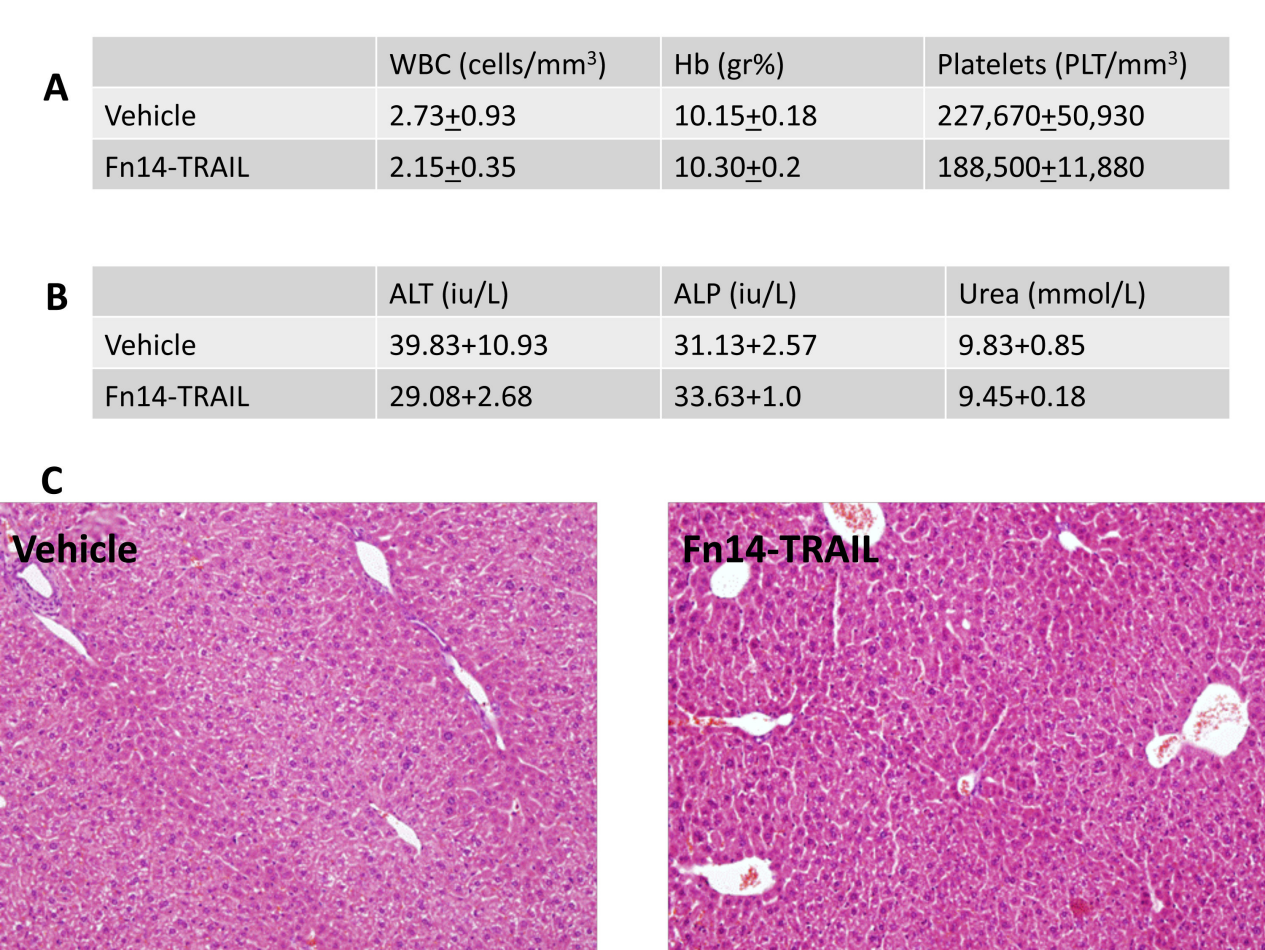
No hepatotoxicty is observed in Fn14•TRAIL treated mice. HepG2 Nude mice were treated daily with subcutaneous injections of 200μg of Fn14•TRAIL (n=4) or vehicle (n=4) for 8 days. One hour after last injection blood counts (**A**) liver enzymes and urea (**B**) were measured. Livers were harvested and H&E stained sections were tested (**C**). Data are presented as mean +/- S.E.

Importantly, no morbidity, liver or renal toxicity were observed in the Fn14•TRAIL-treated mice based upon liver enzymes, histopathological examination and urea measurments as can be seen in Figure 7. 

## Discussion

In the present study, we explored Fn14•TRAIL’s unique properties as an anti-tumor agent. Main findings are: 1) Fn14•TRAIL strongly induces apoptosis in three different HCC cell lines; 2) While HCC cell lines express DR4, DR5 and Fn14 on their surfaces, substantial TWEAK is detected only intracellulary; 3) Non-malignant hepatocyte lines are insensitive to Fn14•TRAIL-induced apoptosis, notwithstanding their similar expression of the aforementioned molecules; 4) Fn14•TRAIL is significantly more effective than soluble Trail, Fn14-Fc or a combination of the two in inducing apoptosis; 5) Fn14•TRAIL's activity can be blocked by Ab directed against TRAIL and Fn14; 6) Fn14•TRAIL effectively inhibits the growth of HCC xenograft tumors in nude mice; and 7) Fn14•TRAIL is well tolerated by mice, with no detectable changes in liver histology. Taken together, these findings establish Fn14•TRAIL’s potential as anti-tumor agent, extending its therapeutic possibilities beyond treatment of autoimmunity. 

Soluble TRAIL has been considered for some time now for its potential as a cancer therapeutic [23–25]. However, some malignant tumors are known to be resistant to the pro-apoptotic effect of soluble TRAIL. While recombinant TRAIL variants, as well as agonist Ab with specificity for the TRAIL receptors DR4 and DR5, were used for inhibiting the growth of various types of malignant cells both *in vitro* and *in vivo* [26–29], others, found the same HCC cell lines under study here, (SK-HEP-1, HepG2 and Huh7), to be highly resistant to TRAIL-induced apoptosis [30,31]. This resistance was notwithstanding their expression of DR4 and DR5. Our observation that Fn14•TRAIL, a fusion protein derivative of this same protein, engenders robust apoptosis of the same malignant cells at extremely low concentrations (less than 3 ng/ml in the case of SK-HEP-1 cells) is especially notable. 

The basis for Fn14•TRAIL’s enhanced pro-apoptotic activity may be several-fold. One possibility is that it stems from the synergy attained by virtue of coordinate blocking of the TWEAK ligand and triggering of TRAIL receptors. However, our repeated observation that the fusion protein is consistently more effective than its soluble components (Fn14 and TRAIL) in combination suggests that there may be yet other explanations for Fn14•TRAIL’s superior activity. One of these explanations revolves around molecular structure, with the possibility that Fn14•TRAIL assumes a higher-order configuration that allows it to function as ’super-TRAIL’. For instance, this could result from stabilization of the TRAIL trimer via TWEAK-induced trimerization of the Fn14 end. TRAIL and other members of the TNF receptor family were shown to be more potent in the trimer form (18-20). 

The Ab-blocking experiments of the present study shed some light on these mechanistic possibilities. Anti-Fn14 Ab completely abrogated Fn14•TRAIL's pro-apoptotic activity. Possible explanation for this key observation is that the Ab interferes with Fn14’s binding to TWEAK, which in turn could impact both higher order structure of the chimeric protein and/or molecular arraying and signaling at the cell surface. 

The variable sensitivity of HCC cell lines to Fn14•TRAIL’s pro-apoptotic activity is of interest. However, we could not correlate this differential sensitivity with the protein and mRNA levels of TRAIL receptors, TRAIL, Fn14 and TWEAK in the targeted tumor cells. This is in agreement with previous reports indicating that wide range of tumors express TRAIL receptors, but these are not correlated with sensitivity to TRAIL–induced apoptosis [11,32,33], and post translation modifications of the receptors, influencing their activity has been proposed to explain this phenomena. We did observe that those HCC cells more sensitive to soluble TRAIL tended to be more sensitive to Fn14•TRAIL. Looking at the intracellular signaling pathways, we found that decreased expression of anti-apoptotic signals in parallel with activation of the pro-apoptotic ones was associated with higher sensitivity to Fn14•TRAIL. Decreased expression of the anti-apoptotic signals was not observed in non-malignant cells. 

The role of TWEAK in this system remains somewhat enigmatic. Whereas surface TWEAK could not be readily detected by immunofluorescence, this protein was readily detectable intracellularly and in conditioned medium. We could not show effect of Fn14•TRAIL on TWEAK:Fn14 signaling pathway in this study. However, most studies unfolding the signaling pathways involved in the Fn14:TWEAK axis were performed with recombinant TWEAK added to the experimental setting [34,35], and this is not the case in our study. TWEAK independent Fn14 signaling have been implicated in some tissues [36], however, it has not been described in HCC cell lines, and therefore it is not expected that Fn14•TRAIL will influence this signaling pathway. 

There is no consensus as to whether it is more beneficial to block as opposed to activate the TWEAK: Fn14 signaling axis in the context of cancer therapeutics. Arguing for blockade are studies indicating the importance of TWEAK in tumor cell survival, resistance to apoptosis and migration [7,8,34,37–39]. Also pointing in this direction is the demonstration that enforced TWEAK over-expression enhances proliferation *in vivo* [40]. This view is consistent with our suggestion of using Fn14•TRAIL as a TWEAK blocker.

On the other hand, there is data in the literature arguing in the other direction, for a potential benefit in activating this pathway. These data encompass findings that TWEAK itself possesses pro-apoptotic activity, either directly through activation of the caspase cascade [41,42], or indirectly via inducing TNF-alpha [35]. The indirect mechanism parallels that associated with other death domain-deficient receptors, such as CD30 and CD40 [43,44] . Also, a recent study has shown that agonistic anti-Fn14 Ab has anti-tumor activity, both *in vitro* and *in vivo* [42]. However, caution is called for in interpreting the latter findings given that, as the authors themselves point out, at least some of anti-tumor activity of the agonistic anti-Fn14 Ab, was mediated by antibody-dependent cell cytotoxicity.

Recently, we reported two other fusion proteins, CTLA-4•FasL and CD40•FasL, with cytotoxic activity against tumor cells [20]. Interestingly, we demonstrated that these proteins function, at least in part, by bridging neighboring molecules on tumor cell surfaces [20,45]. Whether Fn14•TRAIL shares mechanistic features with these other fusion proteins (for example, Fn14 binding to a surface molecule other than TWEAK, or to sub-threshold levels of TWEAK) remains to be determined. However, regardless of how this unfolds, the broader concept that fusion proteins with more than one functional component can be used for tumor cell targeting is now being reinforced, and in this way, opens up a new avenue for devising therapeutics that fit into the personalized onco-medicine paradigm. 
